# The path between socioeconomic inequality and cognitive function: A mediation analysis based on the HAALSI cohort in rural South Africa

**DOI:** 10.3389/fpubh.2023.1011439

**Published:** 2023-03-13

**Authors:** Sianga Mutola, F. Xavier Gómez-Olivé, Nawi Ng

**Affiliations:** ^1^School of Public Health and Community Medicine, Institution of Medicine, Sahlgrenska Academy, University of Gothenburg, Gothenburg, Sweden; ^2^MRC/Wits Rural Public Health and Health Transitions Research Unit (Agincourt), School of Public Health, Faculty of Health Sciences, University of the Witwatersrand, Johannesburg, South Africa; ^3^Department of Epidemiology and Global Health, Faculty of Medicine, Umeå University, Umeå, Sweden

**Keywords:** socioeconomic position (SEP), cognitive health, mediation analysis, inequalities, Agincourt HDSS

## Abstract

**Background:**

Socioeconomic position (SEP) strongly predicts late-life cognitive health, yet the pathways between SEP and cognitive function remain unclear. This study assessed whether and to what extent the association between SEP and cognitive function in the adult population in rural South Africa is mediated by some health conditions, behavioral factors, and social capital factors.

**Methods:**

In this cross-sectional study, we used data from the 2014–15 “Health and Aging Africa: A Longitudinal Study of an INDEPTH Community in South Africa” (HAALSI) cohort, including 5,059 adults aged 40+ years from the Agincourt sub-district in Mpumalanga Province, South Africa. SEP, the independent variable, was measured based on ownership of household goods. Cognitive function, the dependent variable, was assessed using questions related to time orientation and immediate and delayed word recall. We used the multiple-mediation analysis on 4125 individuals with complete values on all variables to assess the mediating roles of health conditions (hypertension, diabetes, obesity, and disability), behavioral factors (leisure physical activity, alcohol consumption, and tobacco smoking), and social capital factors (community's willingness to help, trust, sense of safety, and social network contact) in the association between SEP and cognitive function.

**Results:**

Compared to adults in the poorest wealth quintile, those in the richest wealth quintile had better cognition (β = 0.903, *p* < 0.001). The mediation analysis revealed that health conditions mediated 20.7% of the total effect of SEP on cognitive function. In comparison, 3.3% was mediated by behavioral factors and only 0.7% by social capital factors. In the multiple-mediator model, 17.9% of the effect of SEP on cognitive function was jointly mediated by health conditions, behavioral factors, and social capital factors.

**Conclusion:**

Low socioeconomic position is a significant factor associated with poor cognitive function among adults aged 40 years and above in South Africa. Health conditions mainly mediate the effects between SEP and cognitive function. Therefore, actions to prevent and control chronic health conditions can serve as the entry point for intervention to prevent poor cognitive function among people with low socioeconomic status.

## Introduction

Socioeconomic position (SEP) strongly predicts late-life cognitive health (1, 2). Yet, the mechanistic pathways of the effects of SEP on cognitive function remain unclear, especially among populations in low- and middle-income countries (LMICs). As life expectancy worldwide has been rising since 1950, with a significant increase in the proportion of older adults, there is a need for more research to understand the effect of SEP on cognitive function among the older population (3). The United Nations (UN) estimated that there were 703 million persons aged 65 years or over in the global population in 2019 and that this number would double to 1.5 billion in 2050. Furthermore, the growth of the older adult population has been projected to be higher in low- and middle-income countries (LMICs). For instance, the percentage of the population aged 65 years or over almost doubled from 6% in 1990 to 11% in 2019 in Eastern and South-Eastern Asia and from 5% in 1990 to 9% in 2019 in Latin America and the Caribbean (4). In South Africa, the fertility rate has dropped from 2.7 in 2001 to 2.3 in 2013, while life expectancy has steadily risen from 53 years in 2001 to 60 years in 2014, thereby increasing the population of older adults. The number of elderly persons in South Africa is expected to reach 7 million by 2030, compared to 4 million in 2011. Yet, socioeconomic disparities have remained high in certain areas since the Apartheid regime (5–7).

A large volume of research evidence from high-income contexts shows a strong association between SEP and several health outcomes, including cognitive health among older adults (8–17). From these studies, some factors that influence cognition among older populations have also been documented, such as; biological processes, genetic inheritance, psychological factors, social interaction, childhood deprivation, and SEP (18–20). In Southern Africa, there is scarce research investigating the association between SEP and cognitive function. Some of these studies have shown that low SEP, low education level attained, unemployment, living with HIV/AIDS, childhood deprivation, cardiometabolic diseases, and low social capital are associated with poor cognitive function among older populations (21–25). However, the said studies did not assess the multiple intermediary factors which may mediate the association between SEP and cognitive function. Therefore, there is a need for more research that utilizes models which include multiple factors to fully understand the association between SEP and cognitive function and the possible intermediary factors in the association. This knowledge is essential because the effect of a predictive factor on an outcome is likely to be transmitted by intermediary factors known as mediators (26).

Our study assessed whether and to what extent the association between SEP and cognitive function in the adult population aged 40 years and above in rural South Africa is mediated by health conditions, behavioral factors, and social capital factors. Furthermore, we used the social determinants of health (SDH) framework to explain how inequalities in upstream structural determinants (SEP) influence the more downstream intermediary factors (health conditions, behavioral, and social capital factors) and result in inequalities in health and well-being (cognitive function in this study) (27). The SDH framework also includes a broad range of social conditions in which people live and work, as well as the distribution of money, power, and access to resources related to education and health care, job opportunities, and social contacts (28).

We hypothesized that the SEP association with cognitive function is mediated by health conditions, behavioral and social capital factors among the rural adult population in South Africa and tested the following hypotheses:

1. SEP is positively correlated with cognitive function.
2. SEP is positively correlated with health conditions included (disability, hypertension, diabetes, and obesity) and behavioral factors included (leisure activity, history of alcohol consumption, and history of tobacco smoking). The social capital factors included help, trust, safety, and social network contact.
3. Health conditions, behavioral, and social capital factors positively correlate with cognitive function.
4. Age, sex, marital status, education level, employment status, and childhood health are associated with SEP and cognitive function.

## Methods

### Study design and study site

This study used the baseline data (2014–15) of the “Health and Aging in Africa: A Longitudinal Study of an INDEPTH Community in South Africa” (HAALSI) cohort, which is a representative population-based cohort in the Agincourt sub-district in Mpumalanga Province, in rural North-Eastern South Africa. This paper utilized data from the baseline sample of a random selection of 5,059 adults aged 40 years and above recruited from the 2013 round of the Agincourt Health and Demographic Surveillance System (HDSS) (29). The Medical Research Council (MRC)/Wits Rural Public Health and Health Transitions Research Unit has run the Agincourt HDSS since 1992 (30). The setting is predominantly experiencing high levels of socioeconomic inequalities due to the Apartheid regime (31). Despite minor improvements in socioeconomic conditions in the Agincourt area post-Apartheid, there are still high unemployment levels and disparities in essential services, such as the lack of access to electricity supply, piped water and tarred road coverage (30).

### Data collection

The study sampling and recruitment methods have been described elsewhere (29). In brief, HALSI recruited men and women aged 40 years or above on July 1, 2014, who had lived in the study area for at least 12 months before the 2013 Agincourt HDSS census round. Though HAALSI focuses on older adults, the study recruited adults aged 40 years and over, considering the low life expectancy at birth in SSA, mainly due to HIV/AIDS epidemic (32). The fieldworkers collected data through face-to-face interviews at home using a Computer Assisted Personal Interview (CAPI) system. The interviews covered demographic, education, employment, possession of durable goods and other items like livestock, physical and cognitive functioning, social networks, self-reported health history and non-communicable disease outcomes. The interviews were conducted using xiTsonga, the local language. HAALSI translated all interview materials from English and back-translated them to ensure the instrument's validity and reliability.

### Ethics

The current study used the anonymous HAALSI data available in the public domain and accessible after formal application at https://haalsi.org/data. HAALSI received ethical approvals from the University of the Witwatersrand Human Research Ethics Committee (ref. M141159), the Harvard T.H. Chan School of Public Health, Office of Human Research Administration (ref. C13–1608–02), and the Mpumalanga Provincial Research and Ethics Committee. Once identified, potential participants were informed about the study and asked to provide informed consent in xiTsonga, the local language, or in English. Participants unable to read had a witness and used an inked fingerprint as a signature. The participants' autonomy and privacy were strictly ensured, considering the personal nature of the data they gave. They were also allowed to disengage from the study if they could not continue (29).

### Measurements

#### Independent variable: Socioeconomic position

In our study, we utilized models that included multiple possible intermediary factors in the association of SEP and cognitive function among older adults in post-Apartheid South Africa. In the HAALSI, the household interview included: consumption and expenditures, labor income; business income; government transfers; remittances; housing characteristics, ownership of durable goods, land, livestock and financial assets, and food security. Individual participants were asked about their work status, working hours, income, unemployment, disability income and pensions. The Agincourt HDSS created a wealth index from principal components analysis of household characteristics and ownership of household items, vehicles and livestock (16, 29, 33). The wealth index score was estimated using the random-effects probit model, and the scores were subsequently categorized as the wealth quintiles. Quintile 1 represented the poorest fifth of the population, whereas quintile 5 represented the wealthiest fifth (29). In our study, the wealth quintiles represent SEP.

#### Dependent variable: Cognitive function

HAALSI assessed cognitive function using five validated brief cognitive tests, including items on orientation, immediate and delayed recall, and numeracy, which were adopted from the US. Health Retirement Study (HRS) (21, 29, 34). The adopted HRS tool was harmonized with the Oxford Cognitive Screen (OCS-Plus), a domain-specific cognitive assessment designed for low-literacy settings, especially in LMICS. The tool was then translated and back-translated before piloting it on about half the HAALSI cohort (a sample of 1,402 men and women aged 40–79) to test its validity. The intra-class correlation between similar basic orientation measures in OCS-Plus and HAALSI assessments was 0.79. Therefore, the tool was found to be appropriate for the older population of Agincourt with high levels of illiteracy, where the HAALSI sample was taken from (35). After the pilot study, the following were the five tests used to assess cognition: (1) time orientation: respondents were asked to report the current year, month, and day and the name of the current South African president (one point for each correct answer; four points total); (2) immediate word recall: respondents were asked to recall as many words as possible from 10 words read aloud by the interviewer (one point for each word correctly recalled; 10 points total); (3) delayed word recall: respondents were asked to list the words recalled about 1 minute after an interceding question was asked (one point for each word correctly recalled; 10 points total); (4) counting: respondents were asked to count sequentially from 1 to 20 (one point); and (5) numerical patterns: respondents who successfully counted to 20 were asked to complete the numeric sequence starting from the number two, four, six, and so on (one point). The overall cognitive score ranged from 0 to 26 (21, 23, 24). However, we did not include the last two numeracy tests (counting and numerical patterns) in the present analysis as they are more likely to reflect the individual level of schooling rather than the aging-related cognitive impairment (21). We used the sum of the responses to the first three tests (ranging from 0 to 24) as the continuous cognitive function score. See Supplementary Table A1 for details of questions and their response categories used to define cognitive function in this study.

#### Mediators between SEP and cognitive function

The potential mediator variables included in this study fell under three categories: health conditions, behavioral factors, and social capital factors.

The health conditions included obesity, diabetes, hypertension, and disability. Our study did not include HIV/AIDS in the analysis because we did not have access to the HAALSI data on HIV serostatus, HIV viral load, and the presence of antiretroviral therapy (ART) for HIV-positive patients that required a more extended application. We assessed obesity based on measured height and weight using body mass index (BMI; kg/m^2^) categories which were defined as per standard definitions as follows; obese ≥30 BMI, overweight BMI 25 to <30, normal BMI 18.5 to < 25, and underweight BMI < 18.5 (23). Hypertension was defined as systolic BP ≥ 140 mmHg or diastolic BP ≥ 90 mmHg or if the respondents were currently on antihypertensive medication at the interview. Diabetes was defined as blood glucose ≥ 7 mmol/l (126 mg/dL) in the fasting group (defined as >8 h) or blood glucose ≥11.1 mmol/l (200 mg/dL) in non–fasting samples or self-reported current diabetes treatment at the time of the interview. Individuals with missing fasting information were considered not fasting (23). The respondents were asked to report difficulty or inability to do the following five activities of daily living (ADL): bathing, eating, getting in/out of bed, toileting, and walking across a room. For analysis, a dichotomous variable was generated as a proxy for disability, taking one if the respondent reported difficulty on one or more ADLs (1+ ADL) and 0 otherwise (25).

Behavioral variables included leisure physical activity, history of alcohol intake, and history of tobacco smoking. Leisure physical activity was assessed by asking the respondents if they engaged in vigorous or moderate-intensity physical activities lasting more than 10 min in their spare time. We assessed alcohol consumption by asking whether the respondent was currently consuming an alcoholic drink such as beer, wine, spirits, fermented cider, or traditional alcohol such as thothotho. We also assessed tobacco smoking by asking whether or not the respondent was currently smoking tobacco products such as cigarettes, cigars, or pipes. In this case, we can assume that the wealth index as a measure of SEP is more stable over time and could precede the hypothesized mediators. Additionally, evidence has shown that SEP is one of the many factors influencing a person's alcohol and tobacco use. Despite the ubiquitousness of alcohol and tobacco, their use increases with high SEP because it is considered as a prestigious lifestyle behavior (36).

The social capital variables included the assessment of the community's willingness to help, trust in other members of the community, feeling of safety, and social network contact. Participants were asked if most people in the village were willing to help their neighbors using a Likert scale with categories of strongly agree, agree, disagree, and strongly disagree. We assessed the level of trust in other community members by asking the respondents whether most people in the village could be trusted using the same Likert scale. The responses to the Likert scale were dichotomised to “yes” if the respondents indicated that they strongly agreed or agreed and “no” if they disagreed or strongly disagreed. Community safety was assessed by asking: “In general, how safe is your village?”. The responses (1 = extremely safe, 2 = safe, 3 = not safe, and 4 = extremely unsafe) were dichotomised (3 and 4 recoded to 0 representing “Not safe” and 1 and 2 recoded to 1 representing “Safe”). The participants' social network contact was assessed by asking participants to describe how often they typically interacted with up to seven individuals in person over the past 6 months (1 = every day or almost every day, 2 = a few times per week, 3 = once per week, 4 = a few times per month, 5 = once per month, 6 = a few times in the past 6 months, and 7 = not at all). We generated a new categorical variable, social network contact per week, where 0 = less than once (a few times per month, once per month, a few times in the past 6 months, and not at all), 1 = once or twice per week, 2 = three or more times per week (29).

**Figure 1:**
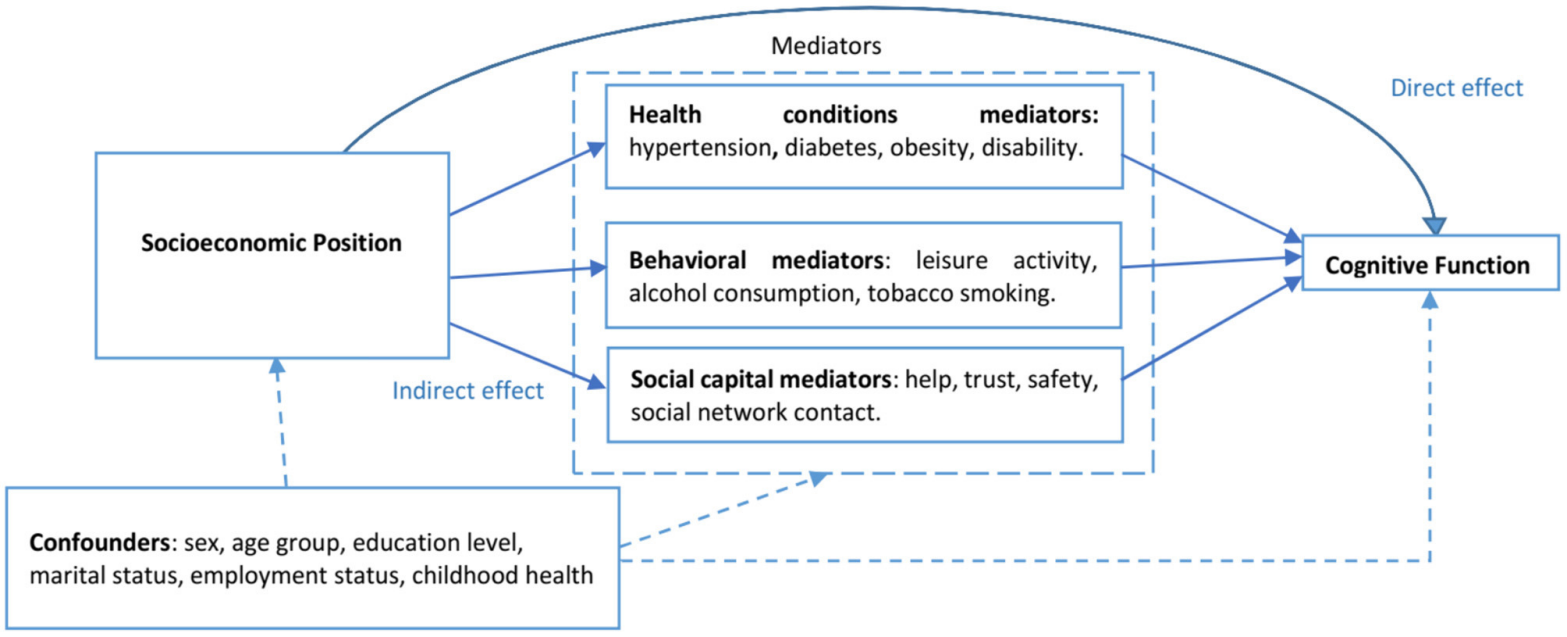
Directed acyclic graph showing the hypothesized mediators in the association between socioeconomic position and cognitive function.

Figure 1 depicts the hypothesized pathways between SEP (wealth quintiles) and cognitive function, demonstrating the possible indirect effect mediated by health conditions and behavioral and social capital mediators.

#### Potential confounders

The potential confounders included in the analysis included age group (40–49; 50–59; 60–69; 70–79; ≥ 80 years old), sex (male or female), marital status (married or unmarried), employment status (employed or unemployed), education (no education or some education), and self-rated childhood health (good or bad). Education level and employment status, which may be highly correlated in other settings, were both included as confounders because the types of jobs in Agincourt are not education-driven or academic-dependent. Agincourt is an underdeveloped rural South Africa where mainly manual and vocational-skills-based employment is available (31). Therefore, this study considered education level and employment status as independent factors.

### Statistical analysis

We assessed for collinearity between the variables and checked for the percentage of missing values for each variable included in our study. After finding high collinearity between literacy and education, we dropped literacy and retained education. In this study, we only included observations with no missing data in all variables (complete case analysis), representing 81.5% of the sample (4,125 individuals out of the total 5,059 individuals in HAALSI). Supplementary Table A2 reports the number and percentages of individuals with complete values and those with missing values on each variable. The missing values ranged from 0.1% (marital status) to 8.1% (diabetes). A total of 4,125 individuals had complete values on all the variables, representing 81.5% completeness. Next, we analyzed descriptive statistics for the socio-demographic characteristics of the participants according to wealth quintiles. We conducted multivariable linear regressions by including groups of mediators and controlling for possible confounders, one at a time and all at once in the full model, in assessing the association between SEP and cognitive function with a statistical significance set at *p* < 0.05.

The next step involved mediation analysis using the multiple–mediator model (37). The multiple-mediator model provided a more accurate assessment of mediation effects than the single-mediator model, which permits only one mediator at a time (26). We used the command “*sem”* in Stata 16.1 to conduct mediation analyses for each of the hypothesized mechanistic pathways of the association between SEP and cognitive function, as depicted in the directed acyclic graph in Figure 1. The mediation analysis procedures involved five steps, with four models analyzed (health conditions model, behavioral model, social capital model, and the full model), as discussed below.

The health conditions model was run, specifying a direct path from SEP to cognitive function while controlling for confounders. The indirect path between SEP and cognitive function, mediated by health conditions while controlling for confounders, was the sum of the products of coefficients for the path from SEP to each of the health conditions (hypertension, diabetes, obesity, and disability) and coefficients for the path from each of the health conditions to cognitive function. The total effect (TE) of SEP on cognitive function was the sum of the natural direct effect (NDE) of SEP on cognitive function and the natural indirect effect (NIE) of SEP on cognitive function. The proportion of the effect of SEP on cognitive function mediated by each set of mediators is the quotient of NIE divided by TE multiplied by 100 (NIE/TE^*^100). The analysis repeated this process for the remaining two mediators (behavioral and social capital mediators).

All the mediators were included in the full model, controlling for confounders. Additionally, we conducted a non-parametric bootstrapping procedure with 500 replications which repeated sampling from the data set and estimated the indirect effect in each resampled data set. By repeating this process 500 times, an empirical approximation of the sampling distribution of the mediator effect in the association between SEP and cognitive function was built and used to construct the 95% confidence intervals (CI) for the indirect effect coefficients. Bootstrapping was achieved by adding the option *vce [bootstrap, reps(500)]* to the *sem* Stata command for each model specified above, following Preacher and Hayes (38) 's multiple-mediator procedures.

## Results

### Characteristics of the participants

The characteristics of the participants in this study, stratified by household wealth quintiles, are presented in Table 1. More people aged 80+ years belonged to the poorest wealth quintile than the richest wealth quintile (13.2 vs. 6.4%). Participants in the poorest wealth quintile were more likely to not work (88.1%) than those in the richest wealth quintile (76.7%). A higher proportion in the poorest wealth quintile (66.1%) had no formal education than participants in the richest wealth quintile (23.1%). More participants from the wealthiest wealth quintile reported being hypertensive (63.1%), diabetic (14.1%), and obese (41.3%) than participants from the poorest wealth quintile (53.0%, 6.7%, and 19.2%, respectively). Regarding social network contact per week, more participants in the poorest wealth quintile (11.5%) reported having no contact per week than those in the richest wealth quintile (8.8%).

**Table 1:** Characteristics of participants based on their wealth quintiles.

	**Wealth quintiles**				
	**1 (poorest)**	**2**	**3**	**4**	**5 (richest)**
	**(*****N** =* **1,046)**	**(*****N** =* **1,001)**	**(*****N** =* **991)**	**(*****N** =* **1,007)**	**(*****N** =* **1,014)**
	***N (%)***	***N (%)***	***N (%)***	***N (%)***	***N (%)***
**Gender**					
Female	544 (52.0%)	546 (54.5%)	541 (54.6%)	550 (54.6%)	533 (52.6%)
Male	502 (48.0%)	455 (45.5%)	450 (45.4%)	457 (45.4%)	481 (47.4%)
**Age group**					
40–49	181 (17.3%)	188 (18.8%)	185 (18.7%)	170 (16.9%)	194 (19.1%)
50–59	298 (28.5%)	265 (26.5%)	256 (25.8%)	285 (28.3%)	306 (30.2%)
60–69	249 (23.8%)	229 (22.9%)	251 (25.3%)	285 (28.3%)	290 (28.6%)
70–79	180 (17.2%)	187 (18.7%)	187 (18.9%)	165 (16.4%)	159 (15.7%)
80+	138 (13.2%)	132 (13.2%)	112 (11.3%)	102 (10.1%)	65 (6.4%)
**Marital status**					
Not married	639 (61.1%)	566 (56.5%)	484 (48.8%)	448 (44.6%)	343 (33.8%)
Married	406 (38.9%)	435 (43.5%)	507 (51.2%)	556 (55.4%)	671 (66.2%)
**Employment status**					
Unemployed	920 (88.1%)	866 (86.9%)	839 (84.7%)	840 (83.7%)	775 (76.7%)
Employed	124 (11.9%)	130 (13.1%)	151 (15.3%)	164 (16.3%)	236 (23.3%)
**Education level**					
No education	690 (66.1%)	554 (55.6%)	445 (45.0%)	383 (38.3%)	234 (23.1%)
Some education	354 (33.9%)	442 (44.4%)	544 (55.0%)	618 (61.7%)	778 (76.9%)
**Childhood health**					
Bad	144 (13.8%)	118 (11.8%)	115 (11.6%)	116 (11.5%)	128 (12.6%)
Good	902 (86.2%)	883 (88.2%)	875 (88.4%)	889 (88.5%)	885 (87.4%)
**History of hypertension**					
No	478 (47.0%)	428 (43.6%)	426 (44.2%)	355 (36.1%)	365 (36.9%)
Yes	539 (53.0%)	554 (56.4%)	538 (55.8%)	628 (63.9%)	625 (63.1%)
**History of diabetes**					
No	898 (93.3%)	838 (90.9%)	809 (88.8%)	806 (87.7%)	802 (85.9%)
Yes	64 (6.7%)	84 (9.1%)	102 (11.2%)	113 (12.3%)	132 (14.1%)
**Obesity**					
Underweight	87 (9.1%)	71 (7.7%)	46 (5.0%)	24 (2.6%)	30 (3.1%)
Normal	433 (45.1%)	384 (41.6%)	346 (37.9%)	320 (34.3%)	236 (24.6%)
Overweight	256 (26.7%)	253 (27.4%)	246 (26.9%)	277 (29.7%)	296 (30.9%)
Obese	184 (19.2%)	216 (23.4%)	275 (30.1%)	313 (33.5%)	396 (41.3%)
**Disability**					
No disability	938 (89.8%)	886 (88.6%)	886 (89.4%)	928 (92.2%)	952 (94.1%)
With disability	107 (10.2%)	114 (11.4%)	105 (10.6%)	78 (7.8%)	60 (5.9%)
**Activity**					
Inactive	313 (30.0%)	315 (31.5%)	322 (32.6%)	356 (35.4%)	322 (31.8%)
Active	731 (70.0%)	685 (68.5%)	667 (67.4%)	650 (64.6%)	692 (68.2%)
**Currently consume alcohol**					
No	695 (66.6%)	741 (74.1%)	775 (78.2%)	811 (80.5%)	865 (85.3%)
Yes	349 (33.4%)	259 (25.9%)	216 (21.8%)	196 (19.5%)	149 (14.7%)
**Currently smoke tobacco**					
No	906 (86.8%)	899 (89.9%)	899 (90.9%)	926 (92.0%)	964 (95.1%)
Yes	138 (13.2%)	101 (10.1%)	90 (9.1%)	81 (8.0%)	50 (4.9%)
**Social network contact per week**					
No contact	111 (11.5%)	95 (10.1%)	108 (11.5%)	108 (11.2%)	87 (8.8%)
One or two	606 (62.7%)	582 (61.9%)	544 (57.9%)	578 (59.8%)	598 (60.7%)
Three +	250 (25.9%)	263 (28.0%)	287 (30.6%)	281 (29.1%)	300 (30.5%)
**People willing to help**					
Unwilling to help	36 (3.6%)	41 (4.2%)	28 (2.9%)	35 (3.6%)	21 (2.1%)
Willing to help	975 (96.4%)	935 (95.8%)	940 (97.1%)	949 (96.4%)	977 (97.9%)
**Trust in community**					
No trust	63 (6.2%)	74 (7.6%)	45 (4.7%)	64 (6.5%)	50 (5.0%)
Have trust	949 (93.8%)	901 (92.4%)	922 (95.3%)	919 (93.5%)	948 (95.0%)
**Community safety**					
Did not feel safe	34 (3.4%)	52 (5.3%)	51 (5.3%)	43 (4.4%)	34 (3.4%)
Felt safe	977 (96.6%)	923 (94.7%)	917 (94.7%)	942 (95.6%)	964 (96.6%)

The total number of observations for each variable does not add to the total sample in each column due to the missing value.

#### Associations between socioeconomic position and cognitive function, adjusting for health conditions, behavioral and social capital factors, and potential confounders

Table 2 displays the five models, with more details in Supplementary Table A3; the basic model, health conditions model, behavioral model, social capital model, and the full model. A positive coefficient for a given category of participants indicates a higher cognitive score than that of the reference group of participants.

**Table 2:** Associations between socioeconomic position and cognitive function, adjusting for health conditions, behavioral and social capital factors, as well as potential confounders.

		**Basic model β (95% CI)**	**Health conditions model β (95% CI)**	**Behavioral model β (95% CI)**	**Social capital model β (95% CI)**	**Full model β (95% CI)**
**Wealth quintiles**						
	1 (poorest)	Ref.	Ref.	Ref.	Ref.	Ref.
	2	0.226 (−0.104, 0.556)	0.129 (−0.217, 0.475)	0.210 (−0.114, 0.533)	0.206 (−0.129, 0.541)	0.0934 (−0.253, 0.440)
	3	0.479 (0.145, 0.812)	0.364 (0.0135, 0.715)	0.480 (0.152, 0.809)	0.411 (0.0724, 0.750)	0.331 (−0.0202, 0.683)
	4	0.596 (0.260, 0.933)	0.323 (−0.0316, 0.678)	0.633 (0.302, 0.965)	0.553 (0.213, 0.893)	0.359 (0.00450, 0.714)
	5 (richest)	1.271 (0.924, 1.617)	1.043 (0.678, 1.409)	1.216 (0.874, 1.559)	1.262 (0.912, 1.612)	1.034 (0.668, 1.399)
**Health conditions**						
**Hypertension**						
	No		Ref.			Ref.
	Yes		0.0978 (−0.135, 0.331)			0.105 (−0.127, 0.337)
**Diabetes**						
	No		Ref.			Ref.
	Yes		−0.368 (−0.731, −0.00399)			−0.408 (−0.769, −0.0464)
**Obesity**						
	Normal		Ref.			Ref.
	Underweight		−0.766 (−1.276, −0.255)			−0.605 (−1.116, −0.0934)
	Overweight		0.521 (0.241, 0.800)			0.461 (0.180, 0.742)
	Obese		0.836 (0.543, 1.129)			0.789 (0.495, 1.084)
**Disability**						
	No disability		Ref.			Ref.
	With disability		−1.061 (−1.522, −0.601)			−0.749 (−1.207, −0.292)
**Behavioral factors**						
**Leisure activity**						
	Inactive			Ref.		Ref.
	Active			1.513 (1.290, 1.736)		1.378 (1.135, 1.621)
**Consume alcohol**						
	No			Ref.		Ref.
	Yes			−0.622 (−0.898, −0.346)		−0.508 (−0.801, −0.215)
**Smoke tobacco**						
	No			Ref.		Ref.
	Yes			−0.579 (−0.983, −0.176)		−0.336 (−0.773, 0.102)
**Social capital factors**						
**People willing to help**						
	Unwilling				Ref.	Ref.
	Willing				0.0223 (−0.605, 0.650)	−0.00169 (−0.666, 0.662)
**Trust in community**						
	No trust				Ref.	Ref.
	Have trust				0.872 (0.387, 1.358)	0.679 (0.165, 1.192)
**Community safety**						
	Felt safe				Ref.	Ref.
	Did not feel safe				−0.836 (−1.373, −0.298)	−0.977 (−1.554, −0.400)
**Social contact per week**						
	No contact				Ref.	Ref.
	Once or twice				0.0390 (−0.319, 0.397)	0.172 (−0.199, 0.543)
	Three +				0.370 (−0.0211, 0.761)	0.460 (0.0549, 0.864)
Constant		9.390 (8.920, 9.860)	9.282 (8.764, 9.800)	8.481 (7.988, 8.975)	9.306 (8.406, 10.21)	8.501 (7.514, 9.489)
Observations		4,800	4,240	4,793	4,664	4,125

All models were adjusted for sex, age group, marital status, education level, employment status, and childhood health.

In the basic model, we observed a gradient of wealth effects on cognitive function. Participants in households in the richest wealth quintile had 1.271 units of cognitive function score higher than their counterparts in the poorest (95% CI: 0.924, 1.617). Furthermore, the older participants had lower cognitive function units than their younger counterparts. Similarly, the results showed a gradient of effects of education on cognitive function, where participants with some education had higher cognitive function than those without formal education.

When we included health condition variables in the model, the effect sizes observed across the different wealth quintiles became smaller. The participants belonging to households in the wealthiest quintile had 1.043 points of cognitive function score higher than those in the poorest wealth quintile (95% CI: 0.678, 1.409). Participants with hypertension had higher cognitive function scores at β = 0.0978 (95% CI: −0.135, 0.331) compared to those with no hypertension, while those with diabetes had lower cognitive function scores (β = −0.368, 95% CI: −0.731, −0.00399) than those with diabetes. We also observed a lower cognitive function score among underweight participants (β = −0.766, 95% CI: −1.276, −0.255) than those with normal weight. The overweight and obese participants showed higher cognitive function scores [β = 0.521 (95% CI: 0.241, 0.800) and β = 0.836 (95% CI: 0.543, 1.129) respectively], than those with normal body weight. The cognitive function score for participants who reported having a disability was −1.061 (95% CI: −1.522, −0.601), units lower than those who did not have a disability.

The participants in the wealthiest wealth quintile were observed to have a higher cognitive function score of 1.216 (95% CI: 0.874, 1.559) than those in the poorest wealth quintile when we included the behavioral variables. The participants who were active in the leisure activity had a higher cognitive function score of 1.513 (95% CI: 1.290, 1.736) than those who were inactive. Those who were currently consuming alcohol had a lower cognitive function score of −0.622 (95% CI: −0.898, −0.346) than those who were not currently consuming alcohol. We also observed that the participants who were currently smoking tobacco had lower cognitive function than those who were not smoking tobacco (β = −0.579, 95% CI: −0.983, −0.176).

The effect sizes observed across the different wealth quintiles in a different model became more prominent when the analysis included the social capital variables. The cognitive function score for participants in the richest wealth quintile was 1.262 units higher than those in the poorest (95% CI: 0.912, 1.612). We also observed a gradient in the effects of social network contact per week on cognitive function, with those with more frequent contact showing a higher cognitive function score than their counterparts with less or no social network contact. The participants who trusted the community had a higher cognitive score (β = 0.872, 95% CI: 0.387, 1.358) than those who distrusted their community.

In the full model with all the mediators, the cognitive function score for the participants in the richest wealth quintile was reduced to 1.034 (95% CI: 0.668, 1.399) units higher than those in the poorest wealth quintile.

#### Assessment of health conditions, behavioral and social capital as mediators in the association between socioeconomic position and cognitive function

Table 3 displays the results of the mediation analysis, reporting the total effects of SEP on cognitive function (TE), as well as the natural direct effects (NDE) and the natural indirect effects (NIE) through the mediators. The results showed a coefficient for the NIE of SEP on cognitive function mediated by the health conditions of 0.06 (95% CI: 0.04, 0.08), behavioral factors of 0.01 (95% CI: −0.01, 0.03), and social capital factors of 0.0017 (95% CI: −0.005, 0.009). These coefficients corresponded to the proportion of the effect of SEP on cognitive function mediated by health conditions of 20.7%, behavioral of 3.3%, and social capital factors of 0.7%. In the full model, the three groups of mediators jointly mediated 17.9% of the effect of SEP on cognitive function.

**Table 3:** Total, natural direct and natural indirect effects of socioeconomic position on cognitive function, with mediation through health conditions, behavioral and social capital pathway.

	**Health conditions model β (95% CI)**	**Behavioral model β (95% CI)**	**Social capital model β (95% CI)**	**Full model β (95% CI)**
Total effect	0.29 (0.20, 0.37)	0.30 (0.22, 0.37)	0.298 (0.22, 0.38)	0.28 (0.20, 0.37)
Natural direct effect	0.23 (0.15, 0.31)	0.29 (0.21, 0.37)	0.296 (0.21, 0.37)	0.23 (0.15, 0.32)
Natural indirect effect	0.06 (0.04, 0.08)	0.01 (−0.01, −0.03)	0.0017 (−0.005, 0.009)	0.05 (0.02, 0.08)
Proportion not mediated	79.3%	96.7%	99.3%	82.1%
Proportion mediated	20.7%	3.3%	0.7%	17.9%

All models were adjusted for covariates. The coefficients for the total effect, natural direct effect, and natural indirect effect were rounded off to 2 decimal points, while the proportion mediated and not mediated were calculated using the unrounded coefficients.

## Discussion

The Social Determinants of Health framework guided the hypothesis generation, selection of variables, and understanding of the mechanistic pathways by which the association between SEP and cognitive function is mediated (27). The study found an association between SEP and cognitive function after controlling for socio-demographic factors, including sex, age, education, employment, marital status, and childhood health, and these findings are similar to the findings by Yang et al. (39). Additionally, our study findings introduce a novel understanding of how the effects of SEP on cognitive function could potentially be mediated. This is important because there is a limited number of studies that have investigated the mechanistic pathways of the effect of SEP on cognitive health, particularly in South Africa where there is a unique socioeconomic context resulting from the Apartheid regime. The findings suggest that an individual's SEP impacts their health status, behavior, and social capital, and therefore, their cognitive health. However, the study results showed that a significant proportion (82%) of the effects of SEP on cognitive function were not mediated by health conditions, behavioral, and social capital mediators and that only 17.9% was mediated. The mediation analysis further showed that health conditions contributed the most to explaining the SEP–cognitive function association, with a mediation effect of 20.7%, followed by behavioral factors of 3.3% and social capital factors of 0.7% when we included the three groups of the mediators independently in the models. The two latter-mediated effects of SEP on cognitive function by behavioral factors [0.01 (95% CI: −0.01, 0.03)] and social capital factors [0.0017 (95% CI: −0.005, 0.009)] were found to be not statistically significant.

Our study found that having diabetes and having a disability resulted in poorer cognitive health. This study's finding is consistent with evidence from Taiwan, where health-related factors were associated with cognitive impairment in older adults (40). However, our study findings showed that hypertension and obesity were positively correlated with cognitive function, consistent with findings in a study among individuals aged 55–85 in Newcastle and Australia (41); as well as in a study in Malawi where poorer cognitive health was associated with lower BMI in the subsistence agriculture context of Malawi's Longitudinal Study of Families and Health (MLSFH), where calorie deficiency is a public health concern (25). In the Malawi study (25), obesity was perceived as a positive outcome and a sign of belonging to higher SEP and having access to an adequate food supply. Additionally, it has been suggested that poor rural populations in Agincourt, involved in agricultural manual work for their livelihoods, have less likelihood of developing sedentary-related obesity (33). However, a systematic literature review of 17 scientific articles revealed impairments in cognition among obese adults (42). Therefore, the high cognitive function among obese participants observed in our study should be understood in the context of existing inequalities in access to wealth and food among post-apartheid rural South Africans. Similarly, we may use this reasoning to understand the higher cognitive function found among hypertensive participants compared to the non-hypertensive participants in this study. All in all, as posited by Hafeman and Schwartz (43), there is a possibility of an interaction between SEP and health status, such that the overall effects of poor health status on cognitive function may be more pronounced in participants with lower SEP compared to participants with higher SEP.

Regarding behavioral factors, this study showed current tobacco smoking and non-engagement in moderate to vigorous physical activity were associated with poorer cognitive function. The adverse impact of tobacco smoking on cognitive health has been widely studied, and the available evidence shows that tobacco alone may contribute to adverse mental and physical health outcomes (44). The effects of moderate to vigorous physical activities on cognition among adults aged 40 years and above have also been documented in other regions and are consistent with our findings (45, 46).

The study results also showed poor cognition among participants currently consuming alcohol, consistent with other research findings, including poor cognitive health, crime, road traffic crashes, high Human Immuno-deficiency Virus (HIV) transmission, and substance abuse (47–49). It is important to note that our analysis did not use other measurements of alcohol consumption, such as the number of units of alcohol and frequency, but only used the “current” questions for tobacco use and alcohol consumption because mediation analysis assumes a time-sequence between exposure, mediator, and outcome variables. Based on our findings, we suggest that public health interventions aimed at preventing cognitive health decline among adults should include tackling alcohol consumption, as also suggested by other researchers (47).

The social capital components analyzed in our study can explain health inequalities observed because they may influence health-related social norms and the diffusion of health-related information (50). This study has confirmed findings from previous research showing that persons with low social support have a higher risk of poor cognitive health (51, 52). Those with higher social network contact enjoyed higher cognitive health (24).

## Strengths and limitations

The strengths of this study include the utilization of comprehensive theoretical and methodological approaches that involve the inclusion of multiple mediators in the mediation analysis, thereby broadening the explanatory spectrum of the SEP—cognitive function association among adults aged 40 years and above. Another significant strength of our study is that it is based on a representative sample from a rural South African district, a world region rarely studied. Additionally, the in-person interviews (home visits) made it possible to include individuals with low literacy and education levels who are generally underrepresented in aging and dementia-related research.

Based on Judd and Kenny (53), we held the following assumptions to assess the mediating effects of the health conditions, behavioral factors, and social capital in the association between SEP and cognitive function: (1) SEP has a direct effect on cognitive function, and (2) SEP has an indirect effect on cognitive function through (a) health status, (b) some behavioral factors, and (c) social capital (see Figure 1). However, caution needs to be exercised when conducting mediation analysis using cross-sectional data (53–55). Mediation analysis using cross-sectional data as performed in several studies (56–58) can be justified if the temporality between the independent, mediator and dependent variables could be assumed, i.e., the hypothesized independent variable predates the mediator, which in turn predates the outcome variable (55). In the HAASI cohort baseline data, SEP was measured by the housing characteristics and ownership of durable goods accumulated over a long time and hence assumed to predate the mediators and cognition status measured in older age (29).

We acknowledge some limitations of this study. Although the study used a comprehensive framework incorporating three pathways and adjusted for several mediator-outcome confounders, there are other potential mediating factors and unmeasured confounders not available in the HAALSI dataset that could bias the estimates reported in this study. Other possible mediators mediating the association between SEP and cognitive function include HIV serostatus, diet quality, working conditions, depression, and life satisfaction. Based on previous studies, the prevalence of HIV/AIDS is relatively high, even among older adults in Agincourt, South Africa (59, 60). Therefore, we recommend further research, including investigating the role of HIV-associated neurocognitive disorder (HAND) (61) in explaining part of the associations and mediation between SEP and cognitive function, in addition to other aging-related neurodegenerative diseases.

Additionally, our study could not infer causal conclusions due to the cross-sectional study design adopted. Finally, the proportion of missing values was relatively high (18.5%), excluding some participants from the analysis. However, the sample size was large enough to support the conclusions. Our sensitivity analysis compared the results of complete case analysis vs. multiply-imputed analysis using three different models: (i) complete case analysis with bootstrapping (the results presented in our paper); (ii) complete case analysis without bootstrapping; and (iii) multiple imputation analysis without bootstrapping. The sem command in Stata software cannot run multiply-imputed data with bootstrapping. The three analyses did not yield different results that invalidated our conclusions; therefore, we decided to keep the analysis based on individuals with complete information on all variables in the analysis.

## Conclusion and implications

The study shows that low SEP is a significant factor associated with poor cognitive function among adults aged 40 years and above in this rural South African setting. However, only 17.9% of the total effects of SEP on cognitive function appeared to be mediated by the three sets of mediators, mainly by health conditions (20.7%). Most importantly, the study findings imply that improving SEP may substantially prevent cognitive decline in later—life. Reducing the inequalities in access to wealth and improving overall physical health among the older population, as a high-risk group, can be effective interventions to improve their well-being and quality of life, precisely their cognitive function.

## Data availability statement

The datasets presented in this study can be found in online repositories. The names of the repository/repositories and accession number(s) can be found below: https://haalsi.org/data.

## Author contributions

SM, FXG-O, and NN conceptualized the research idea. SM and NN analyzed the data. SM prepared the first draft of the paper. FXG-O and NN provided critical inputs on the manuscript. All authors have provided substantial and critical inputs and approved the final draft for publication.
