# Supplementary material for: The path between socioeconomic inequality and cognitive function: A mediation analysis based on the HAALSI cohort in rural South Africa
Source: Front Public Health. 2023 Mar 13;11:1011439. doi: 10.3389/fpubh.2023.1011439 (PMC10040802; doi:10.3389/fpubh.2023.1011439)
Supplement: Supplementary file 1 [file Table_1.DOCX]

**The path between socioeconomic inequality and cognitive function: a mediation analysis based on the HAALSI cohort in rural South Africa**

Sianga Mutola^1^, F Xavier Gómez-Olivé^2,3^, Nawi Ng*^1,4^

1. School of Public Health and Community Medicine, Institution of Medicine, Sahlgrenska Academy, University of Gothenburg, Gothenburg, Sweden.
2. MRC/Wits Rural Public Health and Health Transitions Research Unit (Agincourt), University of the Witwatersrand, Faculty of Health Sciences, Johannesburg, South Africa.
3. INDEPTH Network, East Legon, Accra, Ghana.
4. Department of Epidemiology and Global Health, Faculty of Medicine, Umeå University, Umeå, Sweden.

Corresponding author:

Professor Nawi Ng

Email address: [nawi.ng@umu.se](mailto:nawi.ng@umu.se)

**Supplementary Table A1: Cognitive function measurement questions in HAALSI**

| **Question** | **Response categories** |
| --- | --- |
| **Time Orientation** | |
| 1. What is the year we are in now? | Correct………………………………………….1  Incorrect…………………………………….….2 |
| 1. Can you tell me the month? | Correct………………………………………….1  Incorrect……………………………………..….2 |
| 1. What is the date today? (what day of the month is it today) | Correct………………………………………….1  Incorrect………………………………………...2 |
| 1. Who is the current president? | Correct………………………………….……….1  Incorrect………………………………..……….2 |
| **Immediate word recall** | |
| 1. How would you rate your memory at the present time? Would you say it is excellent, very good, good, fair or poor? | Excellent……………………………….……….1  Very good………………………………..……..2  Good………………………………...………….3  Fair………………………………….………….4  Poor…………………………………...………..5 |
| 1. Overall in the last 30 days, how much difficulty did you have with concentrating or remembering things? | None…………………………………..……….1  Mild……………………………………………2  Moderate…………………………………..…..3  Severe……………………………………….....4  Extreme/cannot do……………………….…….5 |
| 1. Overall in the last 30 days, how much difficulty did you have in learning a new task (for example, learning how to get to a new place, learning a new game, learning a new recipe)? | None………………………………………..….1  Mild……………………………………………2  Moderate………………………………………..3  Severe…………………………………………..4  Extreme/cannot do……………………………..5 |
| 1. Now please let us know the words you are able to recall. | Rice……………………………………………..1 River…………………………………………….2 Doctor……………………………………….….3 Clothes………………………………………….4 Egg………………………..…………………….5  Cat…………………………..………………….6 Bowl…………………………………………….7 Child………………………………..………….8 Hand…………………………………………….9 Book…………………………………………..10  None recalled………………………………….88 |
| 1. Try to remember the words I just read to you. I'll ask you to recall them later. Please count from 1 to 20 | Counted correctly 1 to 20……………..………..1 Cannot count 1-20……………………………...2 Counted incorrectly 1-20……………….………3 |
| **Delayed word recall** | |
| 1. A little while ago, I read you a list of words and you repeated the ones you could remember. Please tell me any of the words that you remember now. | Rice……………………………………………..1 River…………………………………………....2 Doctor…………….…………………………….3 Clothes……………….…………………………4 Egg…………………….……………………….5  Cat……………………..……………………….6 Bowl…………………………………………….7 Child…………………………………………….8 Hand…………………………………………….9 Book…………………………………………..10 |
| 1. [HOW OFTEN DID THE RESPONDENT RECEIVE ASSISTANCE IN ANSWERING THIS SECTION?] | Never……………………….…………………..1  A few times…………………………………….2  Most or all of the time………………………….3 |
| **Numeracy** | |
| 1. I’m going to read you a series of numbers. There will be a blank number in the series that I read to you. I would like you to write down the numbers from left to right and then tell me what number goes in the blank based on the pattern of numbers. 2. . . 4. . . 6. . .BLANK [now please look at the number you just wrote down and tell me the number that goes in the blank]. [The sequence is 2 4 6 8. 8 is the answer we were looking for because, in this example, the numbers increase by 2.] | 8…………………………………………………1 Any number other than 8………………...……..2 Does not know answer…………….……………3 Respondent does not understand instructions……………………………….……..4 |

Note: In this study, we do not include question 9 and 12 in assessing cognitive function as they are more likely to reflect the individual level of schooling rather than the ageing-related cognitive impairment.

**Supplementary Table A2: Percentage of missing data for each variable**

|  | | **Complete values** | | **Missing values** | |
| --- | --- | --- | --- | --- | --- |
|  | | *N* | *%* | *N* | *%* |
| **Variable** | |  |  |  |  |
|  | Gender | 5,059 | 100 | 0 | 0.0 |
|  | Age | 5,059 | 100 | 0 | 0.0 |
|  | Marital status | 5,055 | 99.9 | 4 | 0.1 |
|  | Employment status | 5,045 | 99.7 | 14 | 0.3 |
|  | Education level | 5,042 | 99.7 | 17 | 0.3 |
|  | Childhood health | 5,055 | 99.9 | 4 | 0.1 |
|  | Hypertension | 4,936 | 97.6 | 123 | 2.4 |
|  | Diabetes | 4,648 | 91.9 | 411 | 8.1 |
|  | Obesity (BMI) | 4,689 | 92.7 | 370 | 7.3 |
|  | Disability | 5,054 | 99.9 | 5 | 0.1 |
|  | Leisure activity | 5,053 | 99.9 | 6 | 0.1 |
|  | Consuming alcohol | 5,056 | 99.9 | 3 | 0.1 |
|  | Smoking tobacco | 5,054 | 99.9 | 5 | 0.1 |
|  | People willing to help | 4,937 | 97.6 | 122 | 2.4 |
|  | Trust in community | 4,935 | 97.5 | 124 | 2.5 |
|  | Community safety | 4,937 | 97.6 | 122 | 2.4 |
|  | Social network contact per week | 4,798 | 94.8 | 261 | 5.2 |
|  | Wealth index | 5,059 | 100 | 0 | 0.0 |
|  | Cognitive function | 4,903 | 96.9 | 156 | 3.1 |

¨

**Supplementary Table A3: Associations between socioeconomic position and cognitive function, adjusting for health conditions, behavioral and social capital factors, adjusting for health conditions, behavioral and social capital factors**

|  | | Empty Model  β [95% CI] | Basic Model  β [95% CI] | | Health Conditions Model  β [95% CI] | | Behavioral Model  β [95% CI] | | Social capital Model  β [95% CI] | | Full Model  β [95% CI] | |  |
| --- | --- | --- | --- | --- | --- | --- | --- | --- | --- | --- | --- | --- | --- |
| **Wealth Quintiles** | |  |  | |  | |  | |  | |  | |  |
|  | 1 (poorest) | Ref. | | Ref. | | Ref. | | Ref. | | Ref. | | Ref. | |
|  | 2 | 0.493 [0.121,0.865] | | 0.226 [-0.104,0.556] | | 0.129 [-0.217,0.475] | | 0.210 [-0.114,0.533] | | 0.206 [-0.129,0.541] | | 0.0934 [-0.253,0.440] | |
|  | 3 | 1.071 [0.698,1.443] | | 0.479 [0.145,0.812] | | 0.364 [0.0135,0.715] | | 0.480 [0.152,0.809] | | 0.411 [0.0724,0.750] | | 0.331 [-0.0202,0.683] | |
|  | 4 | 1.403 [1.032,1.774] | | 0.596 [0.260,0.933] | | 0.323 [-0.0316,0.678] | | 0.633 [0.302,0.965] | | 0.553 [0.213,0.893] | | 0.359 [0.00450,0.714] | |
|  | 5 (richest) | 2.633 [2.264,3.003] | | 1.271 [0.924,1.617] | | 1.043 [0.678,1.409] | | 1.216 [0.874,1.559] | | 1.262 [0.912,1.612] | | 1.034 [0.668,1.399] | |
| **Socio-demographic Factors** | |  |  | |  | |  | |  | |  | |  |
| ***Gender*** | |  |  | |  | |  | |  | |  | |  |
|  | Female |  | | Ref. | | Ref. | | Ref. | | Ref. | | Ref. | |
|  | Male |  | | 0.223 [-0.000,0.446] | | 0.456 [0.207,0.704] | | 0.634 [0.393,0.875] | | 0.208 [-0.018,0.434] | | 0.714 [0.449,0.980] | |
| ***Age group*** | |  |  | |  | |  | |  | |  | |  |
|  | 40-49 |  | | Ref. | | Ref. | | Ref. | | Ref. | | Ref. | |
|  | 50-59 |  | | -0.844 [-1.157,-0.531] | | -0.895 [-1.225,-0.565] | | -0.830 [-1.137,-0.523] | | -0.885 [-1.201,-0.569] | | -0.907 [-1.236,-0.579] | |
|  | 60-69 |  | | -1.599 [-1.934,-1.263] | | -1.668 [-2.022,-1.314] | | -1.654 [-1.983,-1.325] | | -1.613 [-1.953,-1.274] | | -1.725 [-2.078,-1.371] | |
|  | 70-79 |  | | -2.532 [-2.911,-2.153] | | -2.542 [-2.945,-2.139] | | -2.508 [-2.881,-2.135] | | -2.543 [-2.928,-2.159] | | -2.525 [-2.930,-2.120] | |
|  | 80+ |  | | -3.927 [-4.374,-3.479] | | -3.425 [-3.919,-2.931] | | -3.832 [-4.273,-3.392] | | -3.963 [-4.415,-3.511] | | -3.410 [-3.904,-2.916] | |
| ***Marital status*** | |  |  | |  | |  | |  | |  | |  |
|  | Not married |  | | Ref. | | Ref. | | Ref. | | Ref. | | Ref. | |
|  | Married |  | | 0.637 [0.411,0.864] | | 0.550 [0.311,0.788] | | 0.541 [0.318,0.765] | | 0.612 [0.376,0.848] | | 0.491 [0.246,0.737] | |
| ***Employment status*** | |  |  | |  | |  | |  | |  | |  |
|  | Unemployed |  | | Ref. | | Ref. | | Ref. | | Ref. | | Ref. | |
|  | Employed |  | | 0.772 [0.470,1.074] | | 0.643 [0.327,0.960] | | 0.714 [0.418,1.010] | | 0.765 [0.462,1.069] | | 0.598 [0.285,0.911] | |
| ***Education level*** | |  |  | |  | |  | |  | |  | |  |
|  | No education |  | | Ref. | | Ref. | | Ref. | | Ref. | | Ref. | |
|  | Some education |  | | 2.104 [1.867,2.342] | | 2.142 [1.894,2.389] | | 2.025 [1.791,2.258] | | 2.105 [1.866,2.345] | | 2.062 [1.815,2.309] | |
| ***Childhood health*** | |  |  | |  | |  | |  | |  | |  |
|  | Bad |  | | Ref. | | Ref. | | Ref. | | Ref. | | Ref. | |
|  | Good |  | | 1.598 [1.276,1.920] | | 1.479 [1.138,1.820] | | 1.568 [1.252,1.883] | | 1.554 [1.230,1.877] | | 1.458 [1.120,1.796] | |
| **Health Conditions** | |  |  | |  | |  | |  | |  | |  |
| ***Hypertension*** | |  |  | |  | |  | |  | |  | |  |
|  | No |  | |  | | Ref. | |  | |  | | Ref. | |
|  | Yes |  | |  | | 0.0978 [-0.135,0.331] | |  | |  | | 0.105 [-0.127,0.337] | |
| ***Diabetes*** | |  |  | |  | |  | |  | |  | |  |
|  | No |  | |  | | Ref. | |  | |  | | Ref. | |
|  | Yes |  | |  | | -0.368 [-0.731,-0.00399] | |  | |  | | -0.408 [-0.769,-0.0464] | |
| ***Obesity*** | |  |  | |  | |  | |  | |  | |  |
|  | Normal |  | |  | | Ref. | |  | |  | | Ref. | |
|  | Underweight |  | |  | | -0.766 [-1.276,-0.255] | |  | |  | | -0.605 [-1.116,-0.0934] | |
|  | Overweight |  | |  | | 0.521 [0.241,0.800] | |  | |  | | 0.461 [0.180,0.742] | |
|  | Obese |  | |  | | 0.836 [0.543,1.129] | |  | |  | | 0.789 [0.495,1.084] | |
| ***Disability*** | |  |  | |  | |  | |  | |  | |  |
|  | No disability |  | |  | | Ref. | |  | |  | | Ref. | |
|  | With disability |  | |  | | -1.061 [-1.522,-0.601] | |  | |  | | -0.749 [-1.207,-0.292] | |
| **Behavioral Factors** | |  |  | |  | |  | |  | |  | |  |
| ***Leisure activity*** | |  |  | |  | |  | |  | |  | |  |
|  | Inactive |  | |  | |  | | Ref. | |  | | Ref. | |
|  | Active |  | |  | |  | | 1.513 [1.290,1.736] | |  | | 1.378 [1.135,1.621] | |
| ***Consuming alcohol*** | |  |  | |  | |  | |  | |  | |  |
|  | No |  | |  | |  | | Ref. | |  | | Ref. | |
|  | Yes |  | |  | |  | | -0.622 [-0.898,-0.346] | |  | | -0.508 [-0.801,-0.215] | |
| ***Smoking tobacco*** | |  |  | |  | |  | |  | |  | |  |
|  | No |  | |  | |  | | Ref. | |  | | Ref. | |
|  | Yes |  | |  | |  | | -0.579 [-0.983,-0.176] | |  | | -0.336 [-0.773,0.102] | |
| **Social capital Factors** | |  |  | |  | |  | |  | |  | |  |
| ***People willing to help*** | |  |  | |  | |  | |  | |  | |  |
|  | Unwilling |  | |  | |  | |  | | Ref. | | Ref. | |
|  | Willing |  | |  | |  | |  | | 0.0223 [-0.605,0.650] | | -0.00169 [-0.666,0.662] | |
| ***Trust in community*** | |  |  | |  | |  | |  | |  | |  |
|  | No trust |  | |  | |  | |  | | Ref. | | Ref. | |
|  | Have trust |  | |  | |  | |  | | 0.872 [0.387,1.358] | | 0.679 [0.165,1.192] | |
| ***Community safety*** | |  |  | |  | |  | |  | |  | |  |
|  | Felt safe |  | |  | |  | |  | | Ref. | | Ref. | |
|  | Did not feel safe |  | |  | |  | |  | | -0.836 [-1.373,-0.298] | | -0.977 [-1.554,-0.400] | |
| ***Social contact per week*** | |  |  | |  | |  | |  | |  | |  |
|  | No contact |  | |  | |  | |  | | Ref. | | Ref. | |
|  | Once or twice |  | |  | |  | |  | | 0.0390 [-0.319,0.397] | | 0.172 [-0.199,0.543] | |
|  | Three + |  | |  | |  | |  | | 0.370 [-0.0211,0.761] | | 0.460 [0.0549,0.864] | |
| Constant | | 10.45 [10.19,10.71] | 9.390 [8.920,9.860] | | 9.282 [8.764,9.800] | | 8.481 [7.988,8.975] | | 9.306 [8.406,10.21] | | 8.501 [7.514,9.489] | |  |
| Observations | | 4823 | 4800 | | 4240 | | 4793 | | 4664 | | 4125 | |  |

95% confidence intervals in brackets

Note: All models were adjusted for sex, age group, marital status, education level, employment status, and childhood health
